# Laparoscopic partial hepatectomy for primary hepatic PEComa: a case report

**DOI:** 10.1186/s12876-026-04884-5

**Published:** 2026-05-02

**Authors:** Bing Liang, Hongjin Shi, Qingbin Zeng, Xin Tie, Kui Long

**Affiliations:** 1 Department of Hepatopancreatobiliary Surgery, The Second Affiliated Hospital of Kunming Medical University, No. 374 Dianmian Road, Kunming, Yunnan 650101 China; 2 Department of Urology, The Second Affiliated Hospital of Kunming Medical University, Kunming, 650101 China; 3 Department of Integrated Traditional Chinese and Western Medicine, The Third Affiliated Hospital of Kunming Medical University, Kunming, 650101 China; 4 Kunming Medical University, Kunming, China

**Keywords:** Liver cancer, Hepatic PEComa, Laparoscopic hepatectomy, Differential diagnosis, Case report, Perivascular epithelioid cell tumor

## Abstract

**Introduction:**

Perivascular epithelioid cell tumors (PEComas) are rare mesenchymal tumors composed of cells exhibiting an epithelioid morphology. These cells typically arrange around small blood vessels (perivascular spaces) and display dual differentiation characteristics of smooth muscle cells and melanocytes. Diagnosis is challenging due to the absence of specific symptoms or tumor markers. This case features a young male patient with a large hepatic PEComa, whose imaging findings resemble those of hepatocellular carcinoma. We have detailed the entire process from diagnosis to treatment to aid in differential diagnosis and surgical planning.

**Case:**

A 31-year-old male patient with no prior medical history underwent a routine health examination 20 days prior to presentation. Although the patient was asymptomatic, ultrasound revealed an incidental hepatic lesion measuring 58 × 50 × 45 mm (maximum diameter 58 mm, or 5.8 cm). The screening center suspected a hemangioma. Subsequently, he presented to our hospital. Comprehensive imaging studies, including ultrasound, computed tomography (CT), and magnetic resonance imaging (MRI), revealed a 58 mm-diameter space-occupying lesion in segments V and VIII of the right hepatic lobe. Imaging findings initially raised suspicion for hepatocellular carcinoma. To minimize surgical trauma and preserve liver function, our team discussed surgical approaches and ultimately decided on a laparoscopic partial hepatectomy. During the procedure, we obtained a specimen for pathological examination. The final histopathological analysis confirmed the diagnosis of a PEComa with undetermined malignant potential. The patient recovered smoothly postoperatively and was successfully discharged.

**Conclusion:**

PEComa has an insidious onset and is rare. Early diagnosis is often challenging, and imaging studies typically show no highly specific findings. Clinical diagnosis frequently relies on biopsy. In terms of treatment, radical resection (R0 resection, i.e., negative margins) represents the definitive therapeutic approach.

## Introduction

PEComa is an extremely rare mesenchymal tumor characterized by a female predominance and predominantly benign behavior, though some cases may exhibit malignant behavior and metastasize. Its incidence is estimated at approximately ≤ 1 per million population [1]. Due to the rarity of this disease, most cases initially undergo surgical treatment. The main feature of the case reported in this article is a young male patient with a large hepatic PEComa, whose imaging findings were similar to those of hepatocellular carcinoma. We have detailed the patient’s clinical presentation, imaging characteristics, histopathological findings, and treatment outcomes to aid in improving the differential diagnosis and surgical planning for this disease.

## Case presentation

### Chief complaints

On January 12, 2026, a 31-year-old male was hospitalized for 15 days after a liver mass was detected during a physical examination 20 days prior.

### History of present illness

A 31-year-old male patient was admitted to our hospital on January 12, 2026, following the discovery of an occupying lesion during a physical examination 20 days prior. He reported no abdominal pain or pressure symptoms and had not received treatment at any other medical facility.

### History of past illness

The patient has no history of surgery, trauma, or other medical conditions.

### Personal and family history

There was no history of hereditary diseases. No family members had similar symptoms.

### Physical examination

On liver palpation, the patient's liver was of normal size and consistency, with smooth margins and surface, and no tenderness.

### Laboratory examinations

Laboratory tests revealed that the patient's alanine aminotransferase (ALT), aspartate aminotransferase (AST), alpha-fetoprotein (AFP), and prothrombin time (PT) were all within normal ranges, with no history of hepatitis B (Table 1). Among other laboratory indicators, only creatinine and triglyceride levels were elevated; however, the causal relationship between these elevated markers and the disease requires further investigation (Table 1).

**Table 1:** The patient’s laboratory data

Parameter, units	Patient	Reference values
Blood counts		
WBC, × 1,000/μL	7.05	3.5–9.5
RBC, × 10,000/μL	622	430–580
Hb, g/L	187	130–175
Platelets, × 10,000/μL	22.8	12.5–35
Biochemistry and coagulation		
Alb, g/L	49	40–55
AST, U/L	20	15–40
ALT, U/L	28	9–50
ALP, U/L	72	45–125
γ-GTP, U/L	19	10–60
T-bil,μmol/dL	15.4	≤ 26
Urea, mmol/L	3.80	3.1–8.0
Cr, mg/dL	0.93	0.64–1.04
Triglycerides,mmol/L	18.7	< 1.7
PT, s	13.1	9–13
Tumor markers		
AFP, ng/mL	2.92	< 8.78
PIVKA-II, mAU/mL	37.22	< 40.00

*CRP* C-reactive protein, *PT* Prothrombin time, *AFP* Alpha-fetoprotein, *PIVKA II* Protein induced by vitamin K absence or antagonist II

### Imaging examinations

**Fig. 1:**
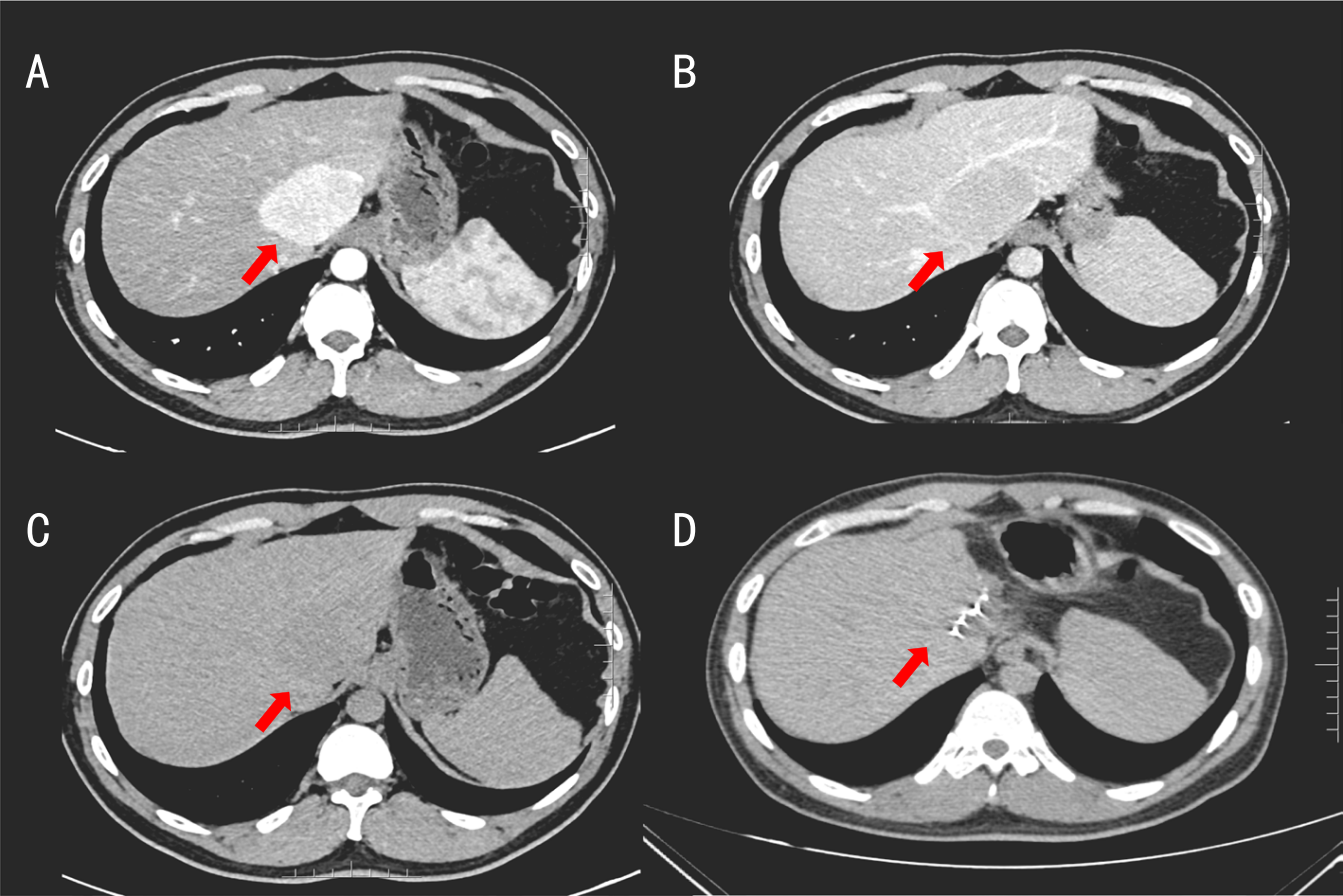
**A** Sagittal-plane enhanced CT arterial phase images (arrow indicates the lesion in segment). **B** Sagittal-plane contrast-enhanced CT venographic phase image (arrow indicates the lesion in segment). **C** Sagittal-plane contrast-enhanced CT delayed phase images (arrow indicates the lesion in segment). **D **Postoperative CT images of the patient (arrow points to the area of the lesion following surgery)

**Fig. 2:**
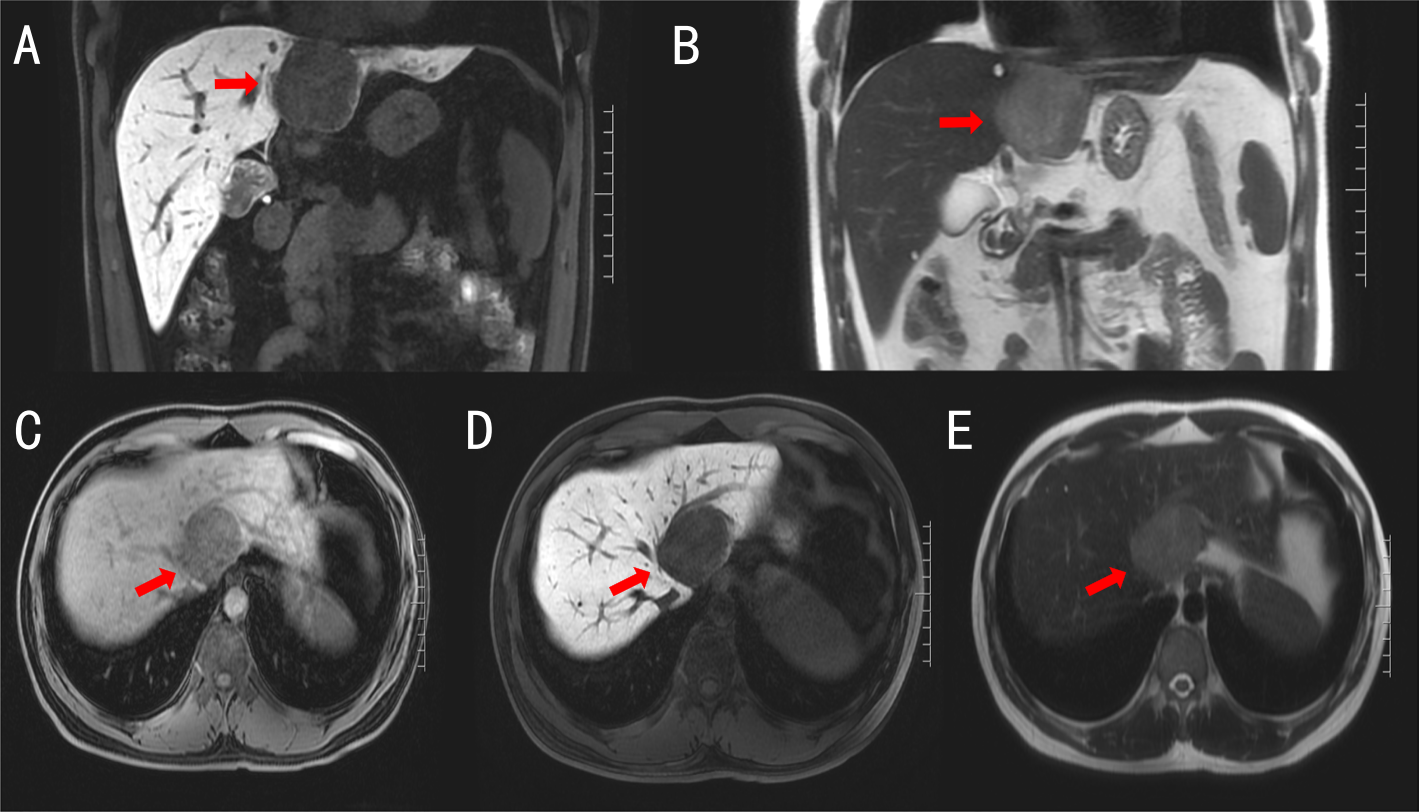
**A** Coronal T2-weighted MRI images (arrow indicates the lesion in segment). **B** Coronal T1-weighted MRI images (arrow indicates the lesion in segment). **C** Sagittal-plane contrast-enhanced T2-weighted arterial phase MRI images (arrow indicates the lesion in segment). **D** Sagittal-plane contrast-enhanced T2-weighted venous phase MRI images (arrow indicates the lesion in segment). **E** Sagittal-plane contrast-enhanced T1-weighted MRI images (arrow indicates the lesion in segment)

CT imaging revealed a slightly hypodense lesion on non-contrast scans, which demonstrated marked enhancement after contrast administration. The tumor involved both hepatic lobes. The lesion appeared as a mass located inferior to segments V and VIII, measuring approximately 58 × 50 × 45 mm (sagittal × axial × coronal planes), with visible cystic components (Fig. 1C). The dynamic contrast-enhanced phase demonstrated marked heterogeneous enhancement (Fig. 1A), with diminished enhancement in the portal and delayed phases (Fig. 1B). The lesion appeared hyperintense on DWI and hypointense on ADC. MRI T1-weighted sequences showed low signal intensity, while T2-weighted sequences revealed high signal intensity (Fig. 1). The number of lesions identified on MRI correlated with the findings of contrast-enhanced CT (Fig. 2).

### Treatment

Given that comprehensive preoperative imaging studies were unable to determine the benign or malignant nature of the tumor or its exact location, our team convened a meeting to develop the optimal treatment plan. The final treatment plan included R0 partial hepatectomy of the affected liver segment.

**Fig. 3:**
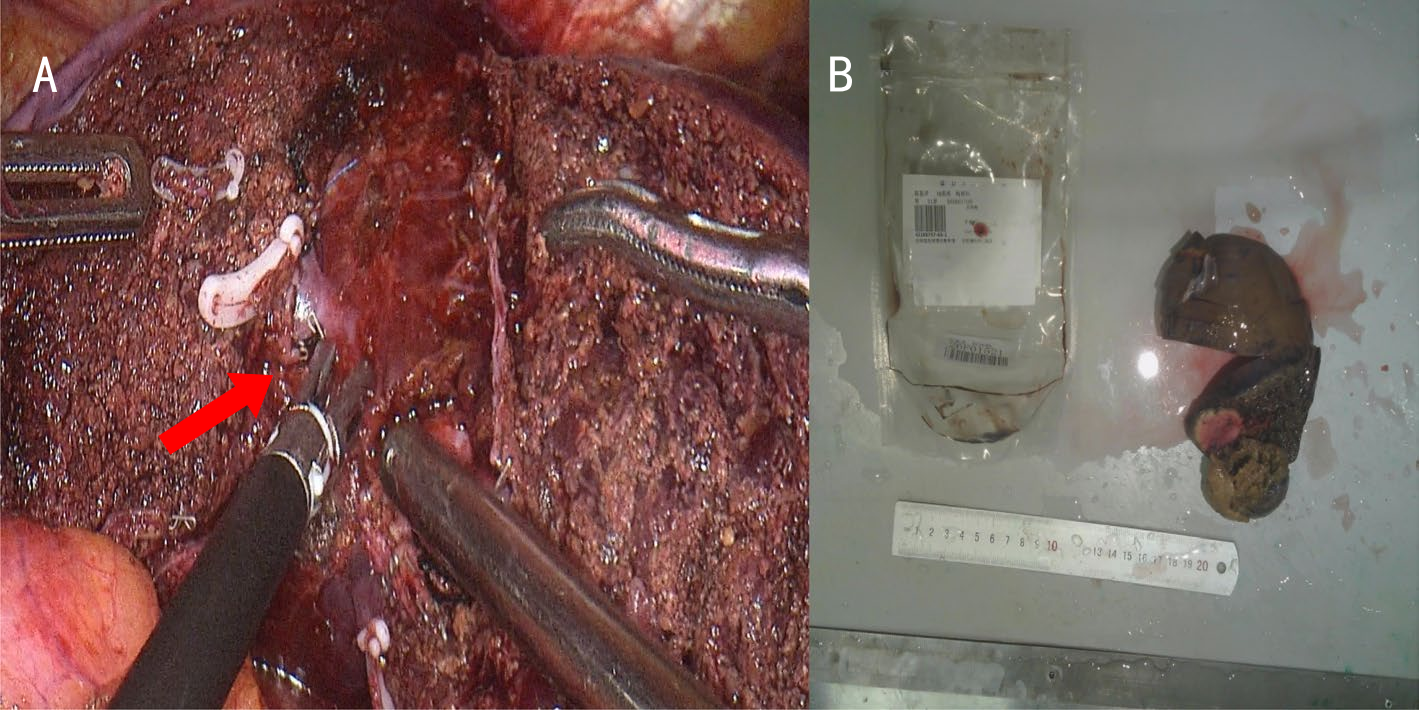
**A** Intraoperative laparoscopic tumor specimen image (arrow indicates the lesion in segment). **B** Postoperative Tumor Specimen Image

We positioned the patient in the supine position and performed laparoscopic partial hepatectomy. After establishing pneumoperitoneum, five trocars were inserted. Indocyanine green combined with a fluorescence-guided laparoscope was used to confirm the location of the lesion and delineate the resection margins. The liver parenchyma was incised using an ultrasonic scalpel, and meticulous hemostasis was achieved with Prolene sutures. The Pringle maneuver was performed intermittently (total clamp time: 40 min). The operative time was 270 min, with an estimated blood loss of 50 mL; no blood transfusion was required. The specimen was retrieved in an endoscopic bag. Pathological examination confirmed negative resection margins (R0), with a minimum margin of 10 mm (Fig. 3).

### Final diganosis

**Fig. 4:**
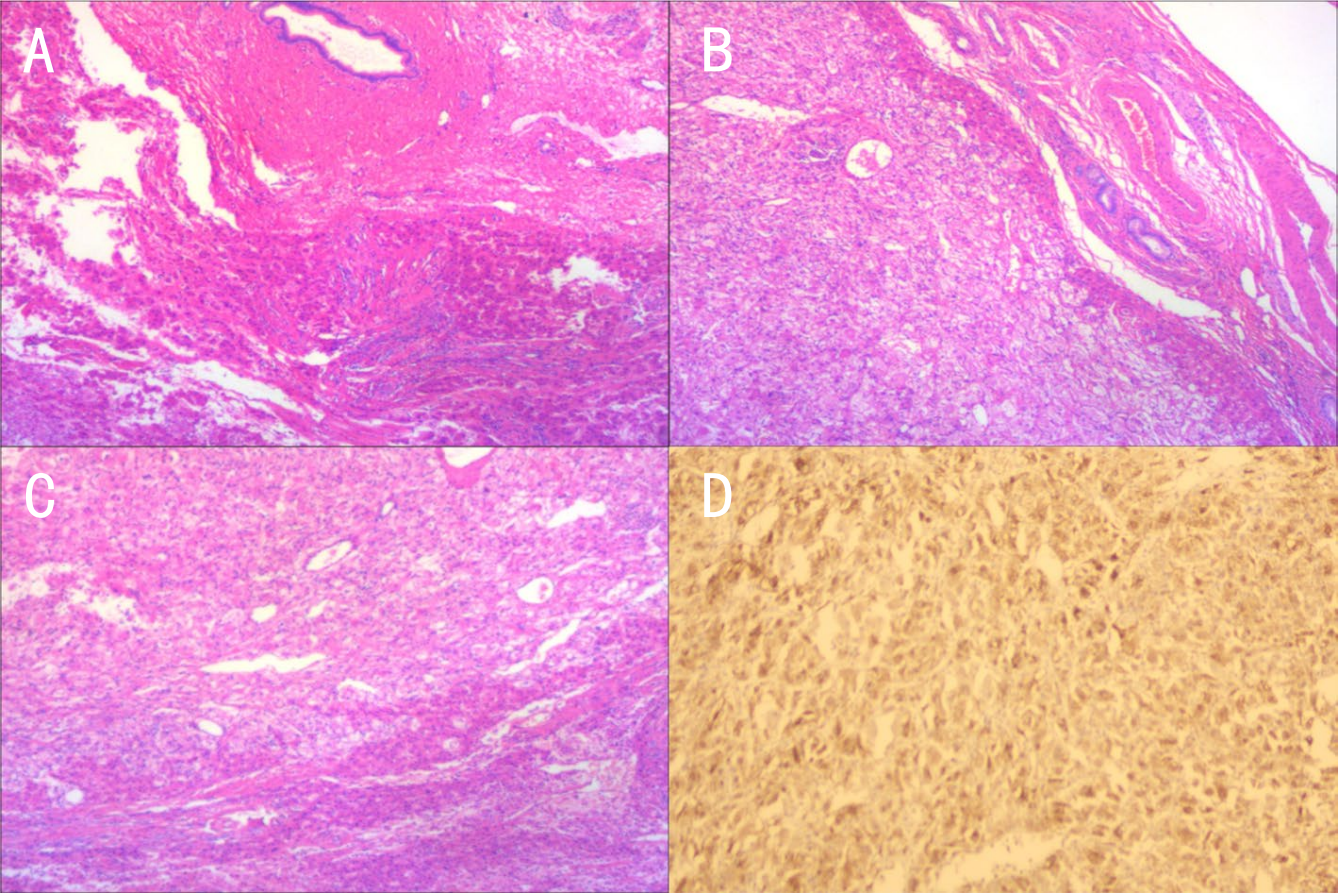
**A**-**C** HE-stained tissue specimens. **D** HMB45 staining of clinical specimen

Postoperative pathological examination confirmed that specimens from both segment V and segment VIII of the liver were PEComas (Fig. 4).

Immunohistochemical analysis demonstrated tumor cell positivity for Glutamine synthetase (GS), p53, β-catenin, Melan-A, HMB45, and CD34. The Ki67 proliferation index was determined to be 5%, with focal positivity for Smooth muscle actin (SMA). Conversely, Cytokeratin (CK) was found to be negative.

### Outcome

On the first postoperative day, the patient exhibited a slight elevation in transaminases, which we attributed to surgical disruption of hepatocytes. A follow-up CT scan performed one week postoperatively demonstrated complete resection of the lesion (Fig. 1D).

## Discussion

Perivascular epithelioid cell tumor (PEComa) is an ultra-rare mesenchymal neoplasm composed of perivascular epithelioid cells that exhibit both melanocytic and smooth muscle differentiation [1]. Epidemiologically, malignant PEComa is classified as an ultra-rare soft tissue sarcoma, with an estimated annual incidence of ≤ 1 case per 1,000,000 population worldwide [1], underscoring its rarity; primary hepatic PEComa is even less common, with the available literature consisting predominantly of isolated case reports and small retrospective series rather than robust population-based datasets [1, 2]. Hepatic PEComa occurs predominantly in women, especially in young and middle-aged adults [2]. The biological behavior of PEComas is heterogeneous, ranging from benign or indolent lesions to tumors with local recurrence, invasive growth, or distant metastasis [3]. To stratify malignant potential, Folpe et al. proposed histopathologic criteria based on tumor size, infiltrative growth, nuclear grade and cellularity, mitotic activity, necrosis, and vascular invasion [4]. In the original Folpe classification, tumors with two or more worrisome features are classified as malignant; lesions larger than 58mm as the sole abnormality, or those showing only nuclear pleomorphism/multinucleated giant cells, are regarded as having uncertain malignant potential; and tumors lacking worrisome features are considered benign [4].

Clinical manifestations of hepatic PEComa lack specificity [5–7]. Most patients present incidentally with hepatic space-occupying lesions during routine examinations or investigations for other conditions [7]. Early-stage patients often experience no significant discomfort [5–7]. As the tumor enlarges, nonspecific symptoms such as abdominal dull pain and bloating may occur due to compression, but overall symptoms remain atypical. Unlike typical hepatocellular carcinoma patients, those with PEComa typically have no history of liver cirrhosis or hepatitis B, and tumor markers like alpha-fetoprotein (AFP) are often normal, as the disease shows no clear association with chronic liver disease [5, 7]. Precisely because of the lack of characteristic clinical manifestations and specific serological markers, hepatic PEComa is frequently misdiagnosed preoperatively as more common liver tumors (such as hepatocellular carcinoma or cavernous hemangioma) [5, 7]. Most cases ultimately require surgical resection and pathological examination for definitive diagnosis.

Imaging findings for hepatic PEComa lack specificity but generally reveal a highly vascular tumor [8]. CT/MRI typically shows marked homogeneous enhancement in the arterial phase, with visible coarse feeding vessels within the lesion [7, 8]. Mild attenuation occurs in the portal venous phase, and the tumor often becomes isodense in the delayed phase. Literature reports indicate that approximately 75% of hepatic PEComas exhibit abnormally thickened vascular patterns within the tumor on imaging, a finding of diagnostic significance for PEComa [7]. This CT example demonstrates rich arterial-phase perfusion with delayed washout, consistent with the characteristics reported in similar cases [9]. However, due to the absence of unique imaging features, the preoperative diagnostic accuracy based solely on imaging is low, with only about 19% of hepatic PEComa cases correctly identified preoperatively through imaging studies [7]. Furthermore, PEComa patients typically lack a history of liver cirrhosis and exhibit normal AFP levels, distinguishing them from most hepatocellular carcinoma cases. Consequently, the preoperative imaging diagnosis of PEComa presents considerable challenges and requires a comprehensive evaluation that incorporates other diagnostic modalities.

Histopathological examination is the primary method for confirming a PEComa diagnosis [10]. Under microscopy, the tumor consists of epithelioid cells that are round or polygonal in shape, with abundant clear or eosinophilic cytoplasm. These cells often arrange in nests, sheets, or even radially around blood vessels, exhibiting varying degrees of nuclear atypia. Immunohistochemistry holds decisive diagnostic value: PEComa tumor cells typically show strong positivity for melanocyte markers (HMB-45, Melan-A), while also expressing smooth muscle markers such as SMA and Desmin [5, 7]. Conversely, epithelial markers like cytokeratin (broad-spectrum CK) and hepatocyte antigen (HepPar-1) are typically negative, aiding in the exclusion of hepatocellular tumors. Studies indicate that over 90% of PEComas express HMB-45 and Melan-A, with more than 80% expressing SMA and other smooth muscle markers. The Ki-67 proliferation index is generally low in benign lesions but markedly elevated in actively proliferating malignant foci. It is important to note that TFE3 gene translocations are observed in some PEComas (more common in younger patients). Immunohistochemistry for TFE3 may show nuclear positivity in these tumors, which is considered indicative of greater invasiveness and poorer prognosis [11]. Therefore, for liver-originating epithelioid tumors of uncertain nature, routine immunohistochemical panel testing, including HMB-45, Melan-A, SMA, Desmin, and TFE3, is essential for definitive diagnosis [11]. The immunophenotype in this case is consistent with the literature, showing strong positivity for HMB45 and Melan-A, while CK and hepatocyte markers are negative, suggesting a non-epithelial tumor. Overall, this case aligns with the common clinical and imaging features described in the literature, but the patient's young age and male gender represent distinct characteristics [5].

Regarding the determination of the tumor’s benign or malignant nature: Imaging measurements indicate a tumor diameter of 58 mm; however, the pathological evaluation revealed no evidence of invasive growth or vascular invasion, the mitotic rate was low, and the Ki-67 index was 5%. According to commonly used risk stratification criteria (including size, mitotic activity, necrosis, and vascular invasion), this lesion exhibits only one feature of concern (primarily related to size); therefore, classification as undetermined malignant potential is most appropriate.

Radical surgical resection (R0 resection) remains the primary and preferred treatment for hepatic PEComa[5–7]. Depending on tumor size and location, anatomical segmentectomy/lobectomy or non-anatomical partial hepatectomy may be selected, but the principle is to achieve complete tumor resection with negative margins whenever possible. For lesions confined to the liver, R0 resection often achieves curative intent: the vast majority of benign hepatic PEComas rarely recur postoperatively and have a favorable prognosis. Due to the lack of preoperative diagnostic specificity for this disease, when malignancy cannot be completely ruled out, the general approach is to actively resect the lesion to confirm the diagnosis and eliminate potential risks. Neoadjuvant sirolimus has been reported to downstage an initially unresectable hepatic PEComa, enabling curative resection [12]. For advanced malignant PEComa, nab-sirolimus demonstrated clinically meaningful and durable responses in the AMPECT trial [13].

The prognosis of PEComa primarily depends on its benign or malignant characteristics [3, 5]. Pathologically, the Folpe criteria are commonly used to assess PEComa malignancy: tumors exhibiting two or more “high-risk” features—including tumor size, nuclear atypia and density, mitotic activity, infiltrative growth, necrosis, and vascular invasion—are classified as ‘malignant’ PEComa [4]. Those with only one feature are considered “malignancy potential undetermined,” while tumors lacking all such features are deemed benign. The vast majority of PEComas meeting benign criteria exhibit low recurrence rates after complete resection and demonstrate a favorable long-term prognosis. In contrast, malignant PEComas often carry a poor prognosis with higher risks of recurrence and metastasis. Malignant PEComa is associated with substantially worse outcomes, with reported disease-related deaths and recurrences across published series [3, 5]. A study encompassing 36 cases of hepatic PEComa revealed: three patients pathologically classified as malignant died within 6 months post-surgery due to tumor progression, while the remaining cases showed no recurrence or metastasis during a median follow-up of 2–4 years [7]. Therefore, patients with high-risk pathological features require enhanced postoperative monitoring and follow-up. For generally benign PEComas, imaging follow-up every 6 to 12 months is recommended postoperatively [3]. In contrast, cases classified as malignant or with undetermined malignant potential necessitate follow-up examinations every 3 to 6 months to detect potential signs of recurrence or metastasis at the earliest possible stage.

The characteristics of this case of primary hepatic PEComa are generally consistent with those reported in the literature: it predominantly occurs in the right liver, is solitary, and exhibits similar immune phenotypes [5, 9]. The main differences lie in the patient's gender and age, suggesting that the diagnosis of PEComa should not be confined to middle-aged women. Due to the non-specific nature of imaging features, the diagnosis of hepatic PEComa primarily relies on pathological immunohistochemistry. Given the potential for malignant transformation in hepatic PEComa, radical surgical resection remains the most effective therapeutic approach [5]. For low-risk small tumors, conservative postoperative observation may be considered, but regular follow-up is essential to monitor for recurrence. Clinicians are advised to enhance awareness of this rare tumor type, include PEComa in the differential diagnosis for similar presentations, and emphasize the importance of long-term postoperative follow-up.

## Data Availability

All data supporting the findings in this case report are included in the article and its supplementary files. Upon reasonable request, de-identified imaging data may be obtained from the corresponding author, subject to institutional policies and patient privacy requirements.
